# A case of olanzapine-associated rhabdomyolysis

**DOI:** 10.1192/bjo.2021.188

**Published:** 2021-06-18

**Authors:** Valentin Skryabin, Michael Zastrozhin, Dmitry Sychev, Evgeny Bryun

**Affiliations:** 1 Moscow Research and Practical Centre on Addictions of the Moscow Department of Healthcare, Russian Medical Academy of Continuous Professional Education of the Ministry of Health of the Russian Federation; 2 Russian Medical Academy of Continuous Professional Education of the Ministry of Health of the Russian Federation; 3 Moscow Research and Practical Centre on Addictions of the Moscow Department of Healthcare

## Abstract

**Aims:**

To describe the case of olanzapine-associated rhabdomyolysis in a 20-year-old patient with a suspected diagnosis of paranoid schizophrenia.

**Method:**

A 20-year-old male Caucasian patient was admitted to the Psychiatric Department with a one-month history of irrational behavior, talking to himself, persecutory delusions, and poor sleep. He was prescribed oral olanzapine at a dose of 10 mg per day. After two days of olanzapine monotherapy, the patient experienced muscle jerks in the legs. Four days after the initiation of olanzapine treatment, he complained about fatigue and weakness in the lower extremities along with myalgia. Physical examination revealed decreased muscle power with no extrapyramidal symptoms. Blood chemistry showed serum creatine kinase (CK) and serum lactate dehydrogenase (LDH) of 9,725 U/L and 843 U/L, respectively, on day four of the therapy. The Naranjo algorithm score of 6 suggested that olanzapine was the probable cause of rhabdomyolysis. A diagnosis of drug-induced rhabdomyolysis was established from the background of blood tests (increased serum CK and LDH levels), clinical presentation (fatigue and weakness in the lower extremities, muscle jerks, and myalgia), and Naranjo algorithm score of 6 for olanzapine. On suspicion of its contribution to rhabdomyolysis, olanzapine was immediately withdrawn. The patient was referred to the intensive care unit. To prevent acute renal failure, high-volume alkaline diuresis was initiated. After consulting a clinical pharmacologist, the patient's primary physician decided to perform a pharmacogenetic test to develop an individualized treatment regimen. Pharmacogenetic test results were interpreted using the PGX2 software (Meditsina LLC, Moscow, Russia). The test revealed that the patient was a homozygous mutant for CYP2D6*4, which corresponds to CYP2D6 PM phenotype. With this in mind, trifluoperazine was prescribed at a daily dose of 10 mg instead of olanzapine as recent data indicate that trifluoperazine is metabolized by CYP1A2 and UGT1A4 instead of CYP2D6. Subsequently, the patient recovered well and was discharged without any nephrological sequelae.

**Result:**

Recent research demonstrates that CYP2D6 is one of the most important isoenzymes implicated in drug metabolism because the CYP2D6 gene is highly polymorphic. Few reports on the association between olanzapine use and rhabdomyolysis have been published to date, and the present case report draws attention to pharmacogenetic testing which allowed the psychiatrist to prescribe another antipsychotic with no risk of rhabdomyolysis.

**Conclusion:**

The presented case demonstrates that pharmacogenetic-guided personalization of treatment may allow selecting the best medication and determining the right dosage, resulting in the reduced risk of adverse drug reactions and pharmacoresistance.

